# Characteristics of minerals and oxide compounds in sediment collected from blood cockle culture areas at Bandon Bay, Thailand

**DOI:** 10.1371/journal.pone.0305061

**Published:** 2024-06-21

**Authors:** Natchanon Jaowatana, Senai Yalçinkaya, Kriengkrai Satapornvanit, Jintana Salaenoi

**Affiliations:** 1 Phuket Marine Biological Center, Ministry of Agriculture and Cooperatives, Phuket, Thailand; 2 Department of Mechanical Engineering, Faculty of Technology, Marmara University, Istanbul, Turkey; 3 Department of Fishery Biology, Faculty of Fisheries, Kasetsart University, Bangkok, Thailand; 4 Department of Marine Science, Faculty of Fisheries, Kasetsart University, Bangkok, Thailand; Hainan University, CHINA

## Abstract

Bandon Bay is a very fertile bay for coastal aquaculture, especially for blood cockles (*Anadara granosa*). Its structural pattern supports the flow of nutrients which directly sent from many rivers resulted the high production capacity of blood cockle at the top level in the country. Besides organic compounds present in sediment, inorganic substances are essential for growth, survival and shell development of blood cockles. A comparative study of minerals and oxide compounds which accumulated in the sediments at eight stations around the cockle culture area was conducted. These stations are located along the estuaries at Tha Thong, Tha Chang, Phum Riang, and Tapi. The proportion of oxide compounds were determinedusing X-Ray Fluorescence (XRF) technique and minerals were analyzed by Atomic Absorption Spectroscopy (AAS). Results showed that sediment characteristics, oxide composition and the amount of minerals among the stations are different from each other. The sediments of the eastern and the western coasts were characterized as crumble clay and muddy sand, respectively. Twelve types of oxide compounds, namely SiO_2_, Al_2_O_3_, Fe_2_O_3_, K_2_O, Cl, MgO, Na_2_O, SO_3_, CaO, TiO_2_, MnO, P_2_O_5_ were found in various quantities, with SiO_2_, Al_2_O_3_, and Fe_2_O_3_ were the fundamental minerals ranging from 85.64–90.82%. Tha Thong estuary in the east coast showed highly significant quantities (P<0.05) of potassium, calcium and manganese compared to the other estuaries.

## Introduction

Bandon Bay is a large cove covering an area of approximately 447 square kilometers. This area is influenced by the monsoon both the southwest monsoon from the Indian Ocean and the northeastern monsoon from the upper Gulf of Thailand that provides heavy rain throughout the year. There are mangrove forests along the edge of the bay and many rivers carry nutrients and organic substances from land flow into the bay causing a river delta. Bandon Bay is a highly fertile area due to the mixing of fresh water from rivers and sea water in addition to adding nutrients and transferring of energy along the food chain. It is suitable for being a food source, and for coastal fishing, aquaculture, refuge, including breeding and nursing young aquatic animals [1]. The overall sea floor area are clay and fine sand texture which is suitable for raising shellfish, especially blood cockles (*Anadara granosa*). From statistics on sea shellfish farming in 2021, Surat Thani Province is the second largest in the country with 328 blood cockle farms, occupying an area of 56,867.37 rai, production volume of 33,526.27 ton with value 3,321.95 million baht, and the large amount of the blood cockle farms is in Chaiya and Kanchanadit District [2].

Bandon Bay is a large bay with a ridge-like landform. The southern boundary of the bay is at the mouth of Tha Thong. There is a jagged coastline that goes straight up to the north and then changes direction to the east. The coastline throughout the bay is shallow and there are mangrove forests growing along the edge of the bay. Sediment deposition areas occur from coastal areas with different elevations. There are many waterways flowing out to the sea. It has the characteristic of deposition of sediments like a river delta, "bird’s foot shape" in various waterways, create a natural embankment extending out into the sea. This makes the coastline wavy and concave like a bird’s foot. It is considered to be the most prominent example of landforms in Thailand [3]. Muttitanon and Tripathi [4] reported on the application of LANDSAT-5 TM satellite images to study the changes in land use of Bandon Bay by considering the visible Red, NIR and MIR wavelength, it was found that the land use has been transformed from vacant land to agricultural area, shrimp farms, mangrove forests, and urban areas. Around the Bay, there are both agriculture and agroindustry such as the fishmeal industry, frozen seafood, canned seafood, crude palm oil, and industries related to rubber. In addition, there are mining concessions including gypsum, dolomite, anhydrite, limestone, kaolin, and ball clay, which can affect natural resources and the environment both directly and indirectly [5, 6].

At present, the area around Bandon Bay has a tendency to deteriorate which is caused by the expansion of communities, houses, agriculture, and industry. Sewage and various chemicals are drained into the water including bringing resources to use over the capacity of the ecosystem to support the quality of water for appropriate habitat and maintain biological activities of living organisms. There were types of shrimp farms, especially white shrimp, *Litopenaeus vannamei*, which production during 2005–2011 averaged 50,175 tons per year and the production in 2012 were 64,820 tons, which was the highest white shrimp production in the country [6]. By the intensive aquaculture, Na nakorn et al. [7] reported the environmental impact of white shrimp culture during 2012–2013 at Bandon Bay generated pollution loading in wastewater and sediment including suspended solids, ammonia nitrogen, organic matter and minerals. The large amount of nutrients caused direct impacts on the coastal aquaculture. However, the overall quality of coastal water in Bandon Bay is still good. The factor that affects most of the water quality and indicated problems such as odor, acidity, alkalinity, dissolved oxygen, nitrate-nitrogen, groups of bacteria and metals, [8] resulted in the blood cockle’s growth rate decreasing and the death rate increasing. The enormous amounts of nutrients and chemicals flowing into the bay caused deterioration of water quality which in turn affected the type and quantity of phytoplankton which are the primary producers in the food chain. This in decreased the potential of blood cockle cultivation areas in Bandon Bay. Sediment is considered a habitat of benthic marine animals and they have a relationship to the marine nutrient cycle. Layers of substances that are fine-sized with organic and inorganic particles, various minerals, dissolved compounds and suspended solids, were sunk gradually and accumulated on the ground as sediment which may be benefit for blood cockles or cause pollution for their growth and survival [9]. In addition, low salinity and long period of low tide are critical problems. Cockles can live in 10–30 ppt salinity, however, it normally likes to bury in the mud about 2.5–3.0 cm depth, but during low tide it sinks into 10–12 cm depth [1] and come up to the surface to find food at high tide. Mud or sand with high amounts of organic substances and gentle currents are required.

Organic substances are vital factors required for the growth and survival of cockles. Inorganic nutrients are another essential element but they must be in the form of a compound that can be taken up by the organism, such as calcium, the main component in shell formation [10]. Therefore, compounds management is important for supplementation to increase absorption, utilization and may increase the value of cockles as well. Sukudom et al. [11] studied organic substances and pH profiles of sediments in Bandon Bay and found that the accumulation of organic substances in sediment varied between seasons and depths. The eastern coast, which was characterized by muddy sediments, had more organic matter accumulation than the western coast, which was sandy soil. The amount of organic matter in sediment was found higher in rainy season than summer and the organic matter varied in content both deep and shallow sediment layer.

Less studies have been done on inorganic elements compared to organic compounds. It is possible that the study of inorganic elements can reveal the relationship between sediment and accumulated nutrients. The relations between types of sediment and trace elements are linked to the association of nutrient exchange between the sediment and the cockles. It is also a guideline for managing and finding areas with suitable environmental conditions for cockle cultivation in order to get the most worthwhile production. Therefore, the study of sediment quality and inorganic substances, especially minerals and oxide compounds, in blood cockle cultivation area in Bandon Bay will present the nature of the sediment condition for explaining the relationship between cockle production in the east and west sides of the bay which are different characteristics. Also used as basic information for considering and deciding on an area that is suitable for growing cockles to have high production in the future.

## Materials and methods

### Sampling location

**Fig 1:**
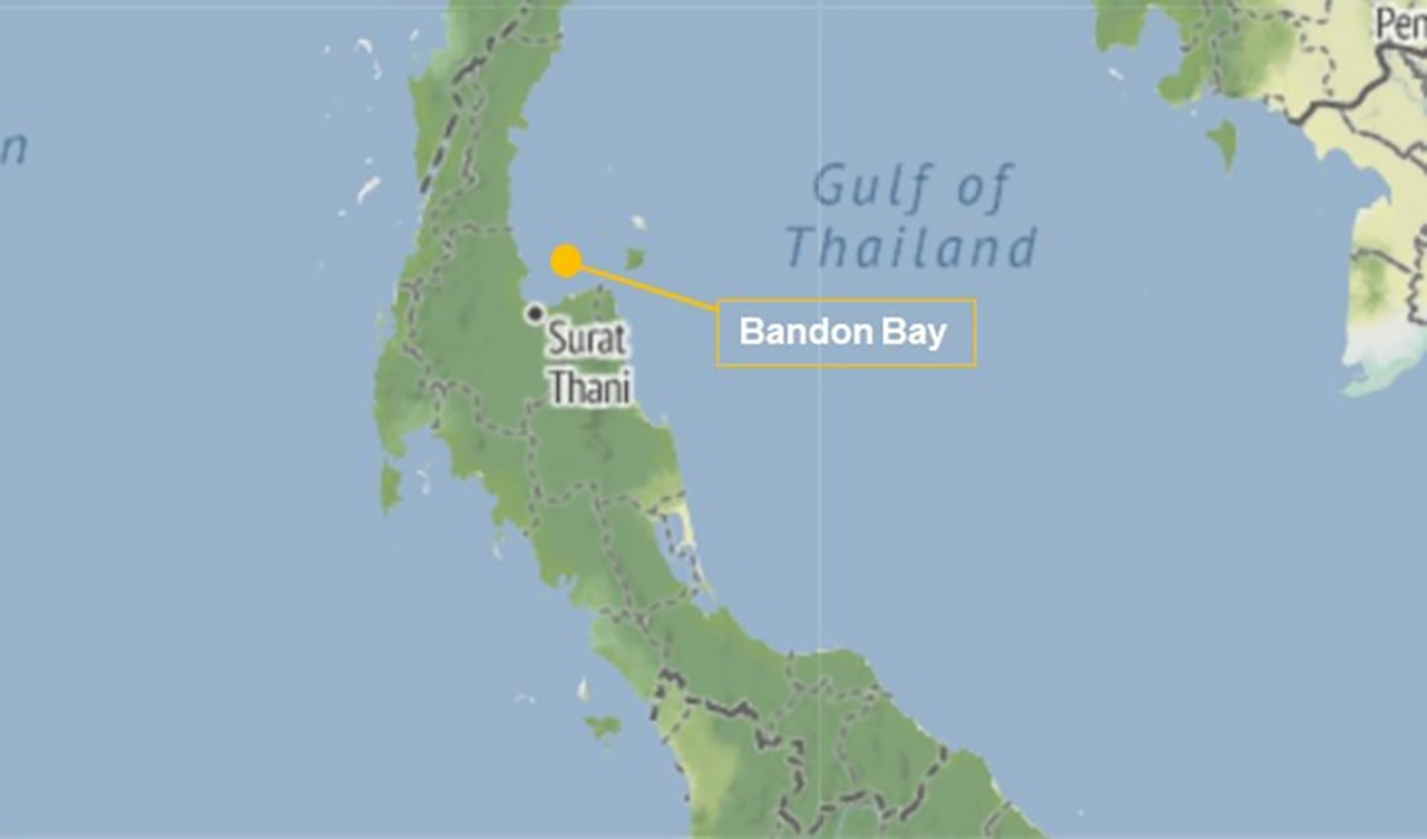
Illustration to explain the location of Bandon Bay, Surat Thani Province. (Source: https://earthobservatory.nasa.gov/map#6/10.337/99.529 [12]).

Sediment samples were collected during the rainy season (August 2019) in 2 parts: the eastern and the western area. The area around the Tapi estuary (St.1) was the center and comparison point. Two stations (St.2 and St. 3) in the east were collected near Tha Thong estuary. Two stations (St.4 and St.5) and three stations (St.6 St.7 and St.8) were collected around Tha Chang estuary and Phum Riang estuary in the west, respectively (Fig 1). For collecting samples in the area, the researchers have directly coordinated with the head of the village to permit collecting samples, facilitate transportation and recommend travel routes. Table 1 presents the coordinates of the sample locations recorded using a global positioning system (GPS).

**Table 1:** Location of eight sampling sites by GPS at Bandon Bay, Surat Thani Province.

Station	Latitute	Longitude	Location
1.	09°16.46.4	099°13.56.4	Tapi estuary
2.	09°14.445	099°24.418	Tha Thong estuary
3.	09°15.469	099°27.316	Tha Thong estuary
4.	09°17.46.8	099°14.28.4	Tha Chang estuary
5.	09°18.39.2	099°16.23.1	Tha Chang estuary
6.	09°21.945	099°16.142	Phum Riang estuary
7.	09°21.274	099°16.441	Phum Riang estuary
8.	09°20.327	099°16.371	Phum Riang estuary

### Sediment sampling

**Fig 2:**
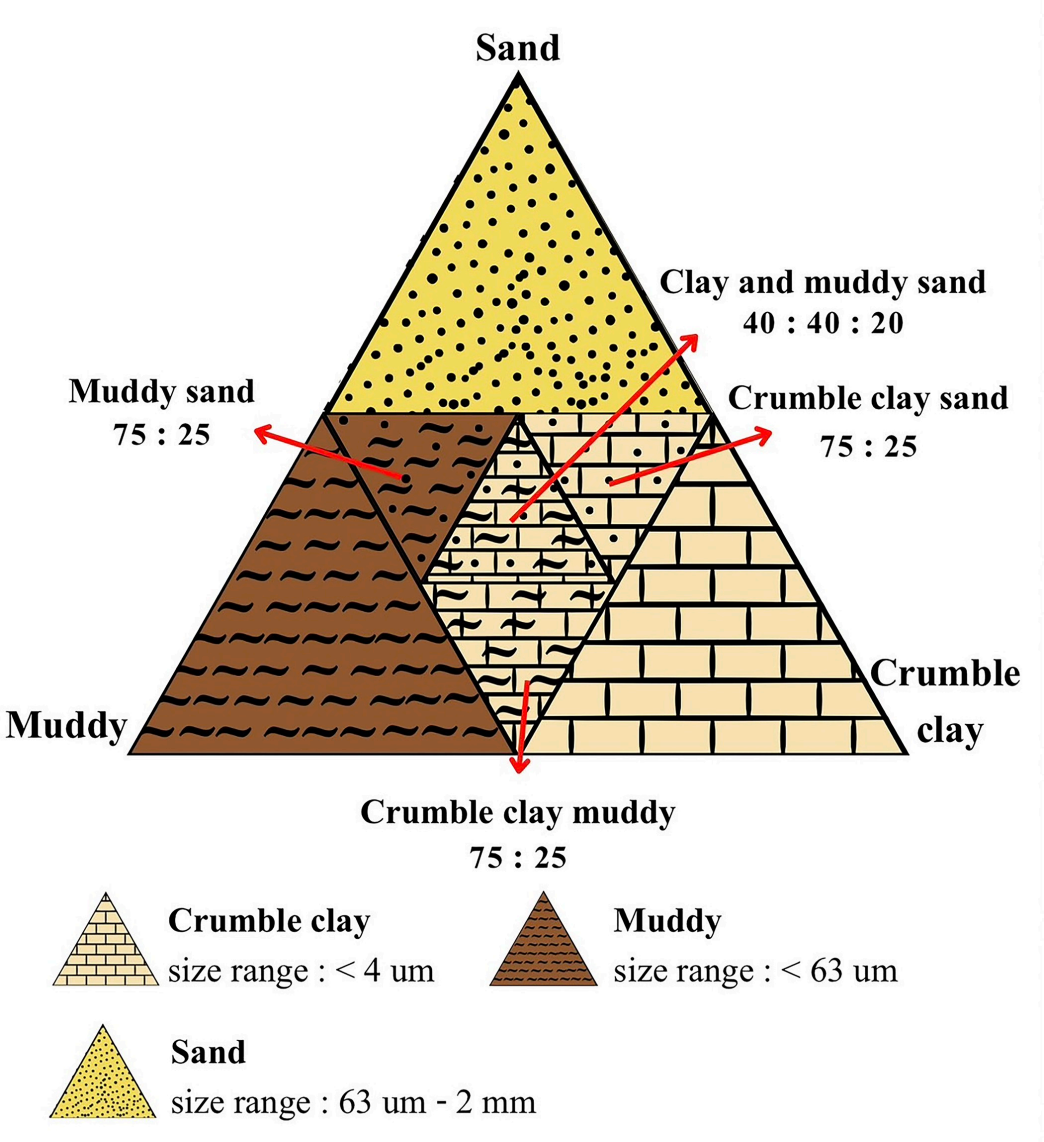
Illustrate to explain the proportion and the component of the sediment.

Sediment samples (in 3 replicates) from the blood cockle farms were collected at each station using hand corers at a depth of 20 cm, stored in a plastic bag which expelled all air out and kept in ice bag. Samples were maintained at -20°C before transferring them to the lab. Precautions to avoid contamination of metals or other compounds, equipment for collecting samples must be made of plastic or uncoated with chromium or other chemicals. The gloves should be changed every time of repeated sample and sampling station. Once the sample is obtained, the sediment must be transferred from the collection device into plastic bag, slowly pour the sample water out of the bag and mix well the sediment before sealing. Every step must be done carefully to prevent loss of sediment samples and the sample bag is labelled with details of sampling for analyzing and interpreting the analysis results correctly. The collected sediment was then dried at 60°C for 24 h, ground thoroughly and kept in test tubes before mineral analysis. Characteristics of sediment samples was described modified from Blair and McPherson [13] as Fig 2.

### Oxide compounds of sediment analysis

The moisture in the sediment was initially analyzed. Soil moisture is the portion of water found in the spaces between soil particles which will indicate the condition of the soil, soil texture, the ratio of inorganic particles and each type of soil has different water holding ability. The sediment samples were dried in an oven at 60°C for 72 h then weighed. The amount of water in the sediment was then calculated [14] as the equation;

Soil Moisture Percentage (%) = [(Wet Soil Weight–Dry Soil Weight) / Dry Soil Weight] x 100

**Fig 3:**
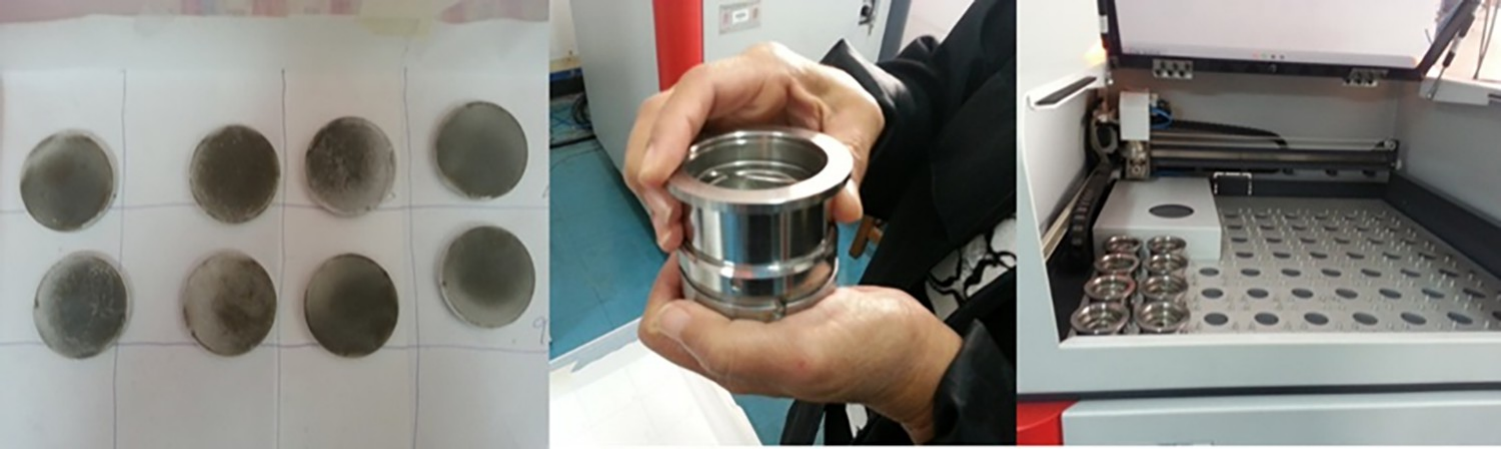
Sediment samples casted with resin to determine mineral proportions with X-Ray Fluorescence Spectrometer.

Then five grams of each dried sample was taken and cast with resin for the determination of the the proportion of minerals using the X-Ray Fluorescence (XRF) technique (Fig 3) with an X-Ray Fluorescence Spectrometer (Bruker AXS, Germany Model: S4 Pioneer, wavelength dispersive, X-Ray Fluorescence (WDXRF) Spectrometry. Voltage/Current 60 kV / 50 mA, Conditions: Range 0.2–20 A (60–0.6 keV), Total resolution 3–100 eV. Typical measurement time 2–10 s per element. Program used: SPECTRA Plus software of the Bruker with the standardless Analysis.

### Analysis of mineral content in sediments

Analysis of mineral content in sediment was done according to Melquiades et al. [15]. By weighing 0.5 g of dry sample (3 replicates for each), digesting with 15 ml 30% HNO_3_, placing on hot plate at 80°C to accelerate the reaction for 4 h. The samples were then added with 18 ml 30% H_2_O_2_ and continued heating for 2 h to digest the organic matter (probably added some distilled water to prevent drying out). After cooling, the sample was filtered with filter paper and adjusted volume to 50 ml with distilled water. The sample was imported into the Atomic Absorption Spectrometer (AAS) to analyze the amount of minerals.

### Statistical analysis

Statistics is a tool that helps in making decisions and planning logically which makes it possible to predict future events close to reality. The analysis results will lead to the preparation of a development plan for cockle production in the coastal area. To compare the differences in mineral content from sediment sampling from stations located in the mouth of the Tapi River. and river mouths on the east and west coasts of Bandon Bay, ANOVA analysis was chosen at a confidence level of 95% (P<0.05), to show significant differences between data. This is considered an appropriate method to analyze the level of variation and multiple comparisons were analyzed using the Turkey HSD method.

## Results and discussion

### Characteristics of sediment

Characteristics of sediment found at stations of Bandon Bay were different from each other. The appearance of sediments in the Tapi estuary (station 1) and the Tha Thong estuary (station 2 and 3 in the east) were crumble clay, those in the west coast in Tha Chang estuary (station 4 and 5) were clay and muddy sand, and the samples in Phum Riang estuary (station 6, 7 and 8) were muddy sand (Table 2). It was clearly shown that the eastern stations had higher clay content than the western stations, while the sediment on the western side was more sandy than clay. This is consistent with Sukudom et al. [11] reported that the eastern area of Bandon Bay had less sandy soil than the western area, but there were highly flexible mud components which was able to hold mineral nutrients well, resulting in water retention and nutrient integration better than in the west where sandy soil was mostly found. And similar to Muttitanon and Tripathi [4] which reported that the two main types of soil in Bandon Bay were muddy and sandy loam, while the muddy soils occurred in the lower tidal range were sulfate-containing soils and most of the surface fresh water draining in Bandon Bay came from the Tapi and Phum Duang rivers.

**Table 2:** Characteristics of sediment samples in estuaries of Bandon Bay, Surat Thani Province.

Coast/Estuary	Station	Characteristics of sediment
Tapi Estuary	1	Crumble clay
Eastern/Tha Thong Estuary	2	Crumble clay
	3	Crumble clay
Western/Tha Chang Estuary	4	Clay and muddy sand
	5	Clay and muddy sand
Western/Phum Riang Estuary	6	Muddy sand
	7	Muddy sand
	8	Muddy sand

Normally, crumble clay is the most fine-textured soil, highly flexible, high ability to hold water and nutrients and difficult to break down. Sandy soil has the largest particle size, is loose holding, easily drains with air ventilation and has less ability to hold water and nutrients. The different sediment textures of the area may affect growth and productivity of benthic animals, especially blood cockles that likes to embed on black or brown fine muddy in the coastal area or the mouth of the river and find food close to their habitat. Most of the organisms in the muddy beach can live in conditions of muddy sediment [16] and from the characteristics of the sediment that has high clay particles will be able to hold on organic matter better than sand. It has been reported that in sediments containing sand will hinder the growth of blood cockles and resulted in slowly growth [17–19]. However, a variety of life on muddy beaches was less than on sandy beaches, but the amount of biomass was higher.

### Inorganic compounds in sediment

**Fig 4:**
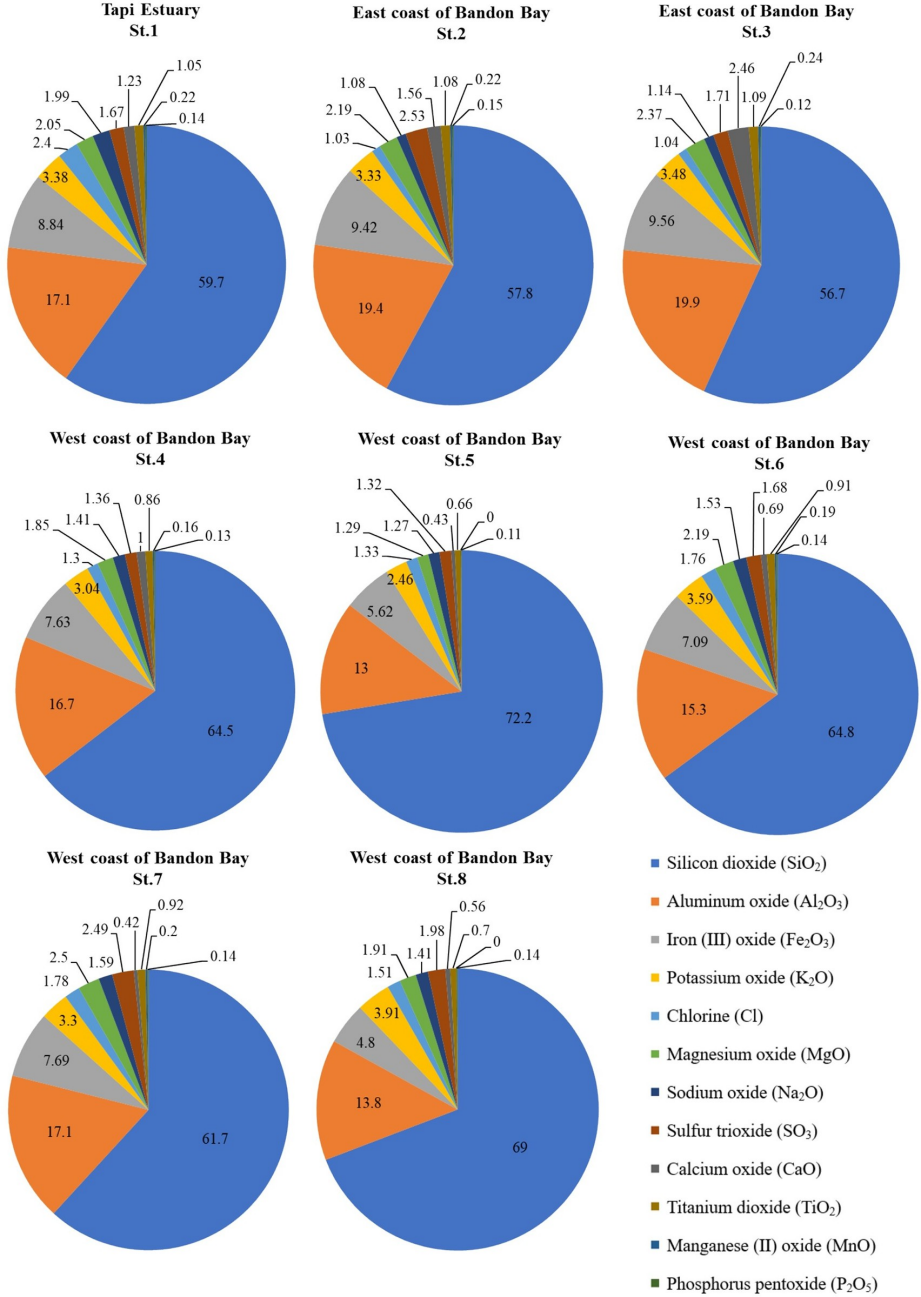
Types and contents of inorganic compounds found in stations at Bandon Bay.

The results of mineral content in the form of oxide compounds in sediments can be divided into 12 types (Fig 4), with silicon oxide (SiO_2_), aluminum oxide (Al_2_O_3_) and ionic oxide (Fe_2_O_3_) being the basic elements with the highest proportions (ranges from 85.64–90.82%) in every station. Station 1 showed the lowest combined proportion at 85.64% and Station 5 had the highest combined proportion at 90.82%. Other mineral components including potassium oxide (K_2_O), chloride (Cl), magnesium oxide (MgO), sodium oxide. (Na_2_O), sulfide (SO_3_), calcium oxide (CaO), titanium oxide (TiO_2_), manganese oxide (MnO), and phosphorus pentaoxide (P_2_O_5_) were presented.

Tapi estuary (St.1) had the highest proportion of Cl (2.40%) whereas Tha Thong estuary had the highest proportion of SO_3_ (2.53%) and P_2_O_5_ (0.15%) and at St.2. St. 3 showed the highest proportion of Al_2_O_3_ (19.90%), Fe_2_O_3_ (9.56%), CaO (2.46%), TiO_2_ (1.09%) and MnO (0.24%). Tha Chang estuary at St. 5 showed the highest proportion of SiO_2_ (72.20%) while Phum Riang estuary found the highest proportion of MgO (2.50%) and Na_2_O (1.59%) at St. 7 and K_2_O (3.91%) at St. 8. SiO_2_ in sediments are the basic components of stations in the western area and had higher contents than Tapi estuary and the eastern area, but the percentage of Al_2_O_3_ and Fe_2_O_3_ in the east was higher than the Tapi estuary and the western.

Hualkasin [20] reported that there were mining industries in the eastern area of Bandon Bay (mainly in Mueang and Don Sak Districts) such as gypsum (CaSO_4_.2H_2_O), anhydrite (CaSO_4_), dolomite (CaMg (CO_3_)_2_, kaolinite (Al_4_(Si_4_O_10_)(OH)_8_), calcite (CaCO_3_) and marble. It is correlated to our results that the high amounts of Al_2_O_3_, CaO, MgO were found in sediment of the east coast. While St. 4, 5, 6, 7, and 8 in the western area presented similar minerals proportion and same type of sandy sediment or sand (SiO_2_) which occur naturally from the decomposition of rocks and are brought together by wind and currents mostly found along rivers or seaside and may be mixed in the sediment.

Jaowatana et al. [21] studied the mineral composition in the sediment from blood cockle culture area in Bandon Bay by X-Ray Diffraction Technique (XRD) and reported that the blood cockles grew well in muddy sediment and found type of minerals in the eastern side similar to the Tapi estuary more than in the western side. The basic minerals found in all stations were quartz (SiO_2_), sanidine (K_0.42_Na_0.58_Ca_0.03_AlSi_3_O_8_), and montmorillonite ((Na,Ca)_0.3_(Al,Mg)_2_Si_2_O_10_(OH)_2_). The other minerals such as forsterite (Mg_2_SiO_4_), calcium oxide (CaO), calcium titanium oxide (CaTiO_3_) and carnallite (KCl.MgCl_2_.6H_2_O) were found in Tapi estuary, forsterite, calcium oxide, Neotocite ((Mn, Mg, Fe) SiO_3_.H_2_O), Iron Oxide (Fe_2_O_3_) and Mica (K.Mg.Fe.Al.Si.O.H_2_O) found in Tha Thong estuary, neotocite and iron oxide found in Tha Chang estuary and orthoclase (K(Al,Fe)Si_2_(OH).H_2_O), iron oxide and olivine (MgFeSiO_4_) found in Phum Riang estuary. Each mineral has different functions such as montmorillonite helps in absorbing organic substances into the sediment, sanidine and orthoclase helps in the attachment of molecules between soil and sediment and mica is the component that gives the shiny sand. Minerals in the nature come from various sources; silicon from quartz [22], magnesium from forsterite and olivine [23], iron from oxide ions [24], calcium from calcium oxide and calcium titanium oxide [25] and potassium from carnallite [26].

The environments play important roles in cockle production [27–30] including the quality of the water, the physical properties of the sediment such as water holding, the exchange of sediment in ions with nutrients in the water and the abundance of minerals in sediments which will affect blood cockle’s life, distribution and food sources and the form of inorganic compounds that can be taken. Hence, phytoplankton such as diatom and some zooplankton are food of cockle [31], therefore, the water quality and the quality of sediments are important and realized. Plongon et al. [32] reported that the average density of phytoplankton was higher in rainy season than summer and the highest amount found in Tapi estuary (8.70x10^6^ units/m^3^) followed by Tha Chang estuary (6.88x10^6^ unit/m^3^), Phum Riang estuary (4.70x10^6^ unit/m^3^) and Tha Thong estuary (2.47x10^6^ unit/m^3^). A total of 83 species of phytoplankton were found and the major group (95%) was diatoms. Silica is the main mineral that be composed in Diatoms which correlated to the present of high silicon dioxide (SiO_2_) in the sediment of this study.

In general, the chemical reaction, the circulation of water and the flow of rivers carrying the nutrients into the sea are considered to be an important replenishment and ensure that nutrients are extremely vital for the survival and growth of phytoplankton. Major nutrients are nitrogen, phosphorus, silicon [33, 34], and the minor nutrients including vitamins and some metals [35, 36]. Plongon et al. [32] showed high concentrations of ammonia (0.155±0.084 mg/l), nitrite (0.017±0.011 mg/l), nitrate (0.063±0.072 mg/l), orthophosphate (0.044±0.048 mg/l), and silicate (0.567±0.269 mg/l) during rainy season which was caused the downward flow of surface water that carry nutrients through community areas, houses, factories, industries, agricultural areas and loading generated from shrimp culture [7].

Our results showed that there were major types of inorganic compounds in the sediment such as silicon dioxide, aluminum oxide and ionic oxide, in every station. While silicate is a very crucial nutrient in the ocean required for certain biota such as diatoms, radiolaria, silicoflagellates, and siliceous sponges. The dissolved silicate in the ocean is converted by various plants and animals into particulate silica (SiO_2_). Six types of silicate consist of Nesosilicates (SiO_4_), Feldspar (aluminum silicate), Quartz (SiO_2_), Mica (aluminum silicate hydroxide), Amphibole (aluminum silicate hydroxide containing magnesium, iron or calcium), Pyroxene (magnesium and iron silicate) and Olivine (magnesium and iron silicate) [37]. Thus, It seemed that Bandon Bay had sufficient silicates to support diatom growth throughout the year. The concentration different form and concentration of particulate silica plays an important role in controlling the growth rate of diatom and the creation of external appearance of diatom and the other groups of marine phytoplankton [38, 39]. Therefore, cockles can grow and increase production well due to the abundance of food such as diatom, phytoplankton and zooplankton.

Plongon et al. [32] reported that in the rainy season, the stations around Tha Thong estuary had high concentrations of ammonia and nitrates, while the stations around Tha Chang estuary and Phum Riang estuary revealed the high content of ammonia and orthophosphate indicated the obtain of phosphate more than other areas. Phosphorus compounds are important macronutrient for promoting the growth of phytoplankton due to the creating energy for cells in the photosynthesis process. It can be stored in large amounts in phytoplankton and rapidly compensate during the shortage of phosphorus in water sources [40] resulted the growth of plants without interruption [41].

### Mineral content in sediments

Determination the amount of minerals including potassium (K), calcium (Ca), magnesium (Mg), sodium (Na) and manganese (Mn) in the sediment was done. It was found that the sediment sample from St. 3 in Tha Thong estuary, eastern area, showed the highest amounts (mg/kg) of K (3,274.0±259.2), Ca (21,860.0±1130.1), Mg (6,449.7±431.4) and Mn (976.7±225.5) (Table 3). For Na, the highest amount (9,134.0±47.5 mg/kg) was found at St. 1 at Tapi estuary. Sediment samples from St. 8 in Phum Riang estuary, western area, showed the least amount (mg/kg) of K (1,237.3±85.1), Ca (384.7±101.4), Na (3.744±417.3) and Mn (26.4±1), while Mg (1,978.3±47.3 mg/kg) was found in the least amount in the St.5 at Tha Chang Estuary. It resulted that Tha Thong estuary in the east coast showed highly significant amounts (P<0.05) of potassium, calcium and manganese compared to the other estuaries.

**Table 3:** Mineral contents found in sediments of stations at Bandon Bay.

Site	Station	Mineral content (mg/kg)				
		K	Ca	Mg	Na	Mn
Tapi estuary	1	2,050.7±57.8^e^	1,279.7±184.6^b^	3,928.0±78.2^c^	9,134.0±47.5^a^	691.0±28.9^b^
Eastern/	2	2,671.7±40.0^c^	410.9±938.9^d^	4,547.0±347.6^b^	6,329.3±272.3^c^	816.0±23.4^a^
Tha Thong Estuary	3	3,274.0±259.2^a^	21,860.0±1130.1^a^	6,449.7±431.4^a^	7,364.9±191.3^b^	976.7±225.5^a^
Western/	4	2,386.0±300.6^d^	1,514.3±232.5^b^	3,685.3±292.2^d^	5,407.7±782.9^c^	629.7±78.7^b^
Tha Chang Estuary	5	1,436.7±60.2^f^	1,079.3±148.8^c^	1,978.3±47.3^e^	4,646.9±144.8^d^	367.7±56.2^c^
Western/	6	2,044.3±16.3^e^	929.0±78.3^c^	4,016.0±904.0^b^	8,125.0±2137.7^a^	499.7±99.4^b^
Phum Reang Estuary	7	2,857.0±162.2^b^	513.0±64.1^d^	6,181.0±138.6^a^	7,143.1±141.9^b^	723.0±196.8^b^
	8	1,237.3±85.1^g^	384.7±101.4^d^	3,373.7±345.7^d^	3,744.0±417.3^e^	26.4±1.6^d^

**Note**: Superscript alphabets in the same column showed statistically significant differences (P<0.05).

It is noticed that sediment samples in the stations at eastern area (mainly St. 3) contained more minerals than those in the western area due to the nature of muddy sediment that nutrient accumulation is easier than the sandy area. The station near the estuary has higher amount of nutrients than the area far away from the river mouth. It was shown that St. 4 (the mouth of the Tha Chang River) has higher amount of Ca compared to St. 6, St. 7 and St. 8 (the mouth of the Phum Riang River), but less content of magnesium and sodium than St. 6 and St. 7. It was also found that area without activities will have lower mineral content than areas with utilization such as St. 8 was near the river channel where no cockle cultivation resulted of less nutrient accumulation compared to St.6 and St. 7.

The Tapi estuary, the large basin and the longest river in the southern region where the most types of minerals were found, showed types of minerals similar to the eastern area compared to the western area. It is resulted from agricultural activities along both sides of the Tapi river that causes the flow of minerals from the agricultural sector into the area, same as the Tha Thong estuary where there is the growth of industry and agriculture such as shrimp farming. The population (88%) in Kanchanadit District engaged in agriculture, fishing and small agricultural industry.

The function of minerals studied (K, Ca, Mg, Na and Mn) are related to the composition and various roles in blood cockles, such as calcium is the main substance in the formation of shells and muscle contraction [42], magnesium and manganese play roles in shell building [43] and potassium and sodium control muscle and nervous function and balance water in the cells [44]. It is related to Boonyuen et al. [45] which found that blood cockle shells consisted of calcium carbonate (CaCO_3_) (95–97%) as calcite form in the prismatic layer, which was the strongest layer. The other components were calcium phosphate (Ca_3_(PO_4_)_2_), magnesium phosphate (Mg_3_(PO_4_)_2_, magnesium silicate (MgSiO_3_) magnesium carbonate (MgCO_3_) and conchiolin protein. Cockles like to bury themselves on muddy or muddy beaches. They come to the surface when the tide is high to find food and bury themselves beneath the surface when the tide is low. Suitable environment for raising is considered an important factor to make cockle farming successful consisting of source of seeds, type of rearing area that must be a smooth mud beach, smooth wind waves, weak water current and the substrate should be fine mud or clay mixed mud and without accumulation of debris mangrove leaves. The water depth and salinity range should be concerned and the rearing area is not influenced by wastewater [1]. Cockles can find food such as phytoplankton or zooplankton in the water and in the sediment. Therefore, the abundance of minerals, organic and inorganic compounds is of paramount importance. Minerals are used for cockles’ external structure and physiological activities within cells, and for phytoplankton or zooplankton growth. The abundance of minerals means the health of the shellfish and investment results as well.

The characteristics of the sediment and minerals are related hence organic matter produced by phytoplankton is one of the main sources of organic material found in marine sediments. It was found that generally more than 90% of the total sediment organic matter could not be separated from the mineral matrix. The adsorption of organic matter to the mineral surface may have an important influence on organic matter retention [46]. And the adsorption of organic carbon to the sediment surface can also affect the availability of organic matter to benthic animals (such as cockles) and bacteria [47] and affect the abundance and distribution of organic substances in marine sediments. Mechanisms that play a role in the adsorption of organic matter to mineral surfaces in marine sediments include ligand exchange, cation bridge, ion and cation exchange, Van der Waals’ reaction and hydrophobic effects [48].

Cockle cultivation in Southeast Asia especially in Thailand, Malaysia and Indonesia has been occurred around for nearly 100 years and most of the farming practices are quite similar. There are 2 types, traditional and development raising. For traditional, it is raised on the sea coast by collecting seeds from natural and grow in appropriate areas. It takes 1–2 years to be raised and then harvested and sold. For the development raising, farming has developed into raising cockles as a large-scale business. Seeds are purchased and sold to small entrepreneurs. Cockle farming is a business operation that does not require much maintenance. It is no need to feed. The important thing to prepare is to procure the seeds. However, factors such as water quality, physical organic and inorganic parameters are quite difficult to control. This is because of large area and is affected by uncontrollable sources. The suitable area or raising place are considered an important factor that will make the career of cockle farming successful [29, 31, 49, 50].

## Conclusion

It is noticed that the amount of minerals accumulated in the sediments is likely to be directly proportional to the production produced in the area. The fertile area will help supporting the growth and the productivity of blood cockles exactly correlated to the results. That is, the contents of minerals deposited in the sediment in the eastern side of Bandon Bay showed the higher accumulation than the western and the Tapi estuary. It seems that the yield from the east coast is therefore likely to be higher than the west coast or Tapi estuary, which is consistent with the report of Department of Fisheries [2] which proposed that the productivities of blood cockle in the eastern side higher than those in the western side due to the fertility and the characteristics of sediment that blood cockle can consume food from. Sediment of crumbly clay in the eastern showed the suitable habitat better that muddy sand in the western.

## Supporting information

S1 File (PDF)
