# Supplementary material for: Characteristics of minerals and oxide compounds in sediment collected from blood cockle culture areas at Bandon Bay, Thailand
Source: PLoS One. 2024 Jun 21;19(6):e0305061. doi: 10.1371/journal.pone.0305061 (PMC11192381; doi:10.1371/journal.pone.0305061)
Supplement: S1 File — (PDF) [file pone.0305061.s001.pdf]

Raw data of oxide compounds in sediment analyzed with X-ray Fluorescence Spectrometer (XRF)

The X-Ray Fluorescence (XRF) technique is based on the principle of absorption and release of energy by materials. When a material is exposed to X-rays, the elements in the sample absorb the X-rays and exudes energy, called Fluorescence. The energy value depends on the type of elements in the sample material and makes it possible to classify what elements are in that material. It can be analyzed both quantitatively and qualitatively. The technique provides fast, high precision, accurate analysis results and does not destroy the object being tested. However, the operating costs are quite high. Therefore, this instrument is chosen to analyze the amount of oxide compounds in sediment for the accuracy and precision of the information. For this analysis, a representative sample of sediment was selected without replication. The data presented in the manuscript is therefore raw data.

Raw data of mineral content in 8 stations of Bandon Bay (3 replications)

Station	Sample no.	K mg/kg	Ca mg/kg	Mg mg/kg	Na mg/kg	Mn mg/kg
Station 1	s-1-1	2057.0	1190.0	3861.0	9102.2	673.0
	s-1-2	1990.0	1157.0	3909.0	9189.4	677.0
	s-1-3	2105.0	1492.0	4014.0	9113.2	725.0
Station 2	s-2-1	2712.0	3056.0	4146.0	6138.2	789.0
	s-2-2	2671.0	4412.0	4762.0	6208.7	829.0
	s-2-3	2632.0	4859.0	4733.0	6641.1	830.0
Station 3	s-3-1	3135.0	21000.0	6717.0	7579.0	847.0
	s-3-2	3114.0	23140.0	6680.0	7304.6	846.0
	s-3-3	3573.0	21440.0	5952.0	7211.0	1237.0
Station 4	s-4-1	2730.0	1627.0	3739.0	6101.6	687.0
	s-4-2	2174.0	1669.0	3370.0	5562.7	662.0
	s-4-3	2254.0	1247.0	3947.0	4558.9	540.0
Station 5	s-5-1	1372.0	977.0	2018.0	4697.9	418.0

	s-5-2	1447.0	1250.0	1991.0	4483.5	378.0
	s-5-3	1491.0	1011.0	1926.0	4759.2	307.0
Station 6	s-6-1	2062.0	955.0	3906.0	8698.8	570.0
	s-6-2	2030.0	991.0	3172.0	9917.2	543.0
	s-6-3	2041.0	841.0	4970.0	5758.9	386.0
Station 7	s-7-1	2992.0	477.0	6056.0	7238.3	504.0
	s-7-2	2902.0	475.0	6330.0	7211.0	885.0
	s-7-3	2677.0	587.0	6157.0	6980.0	780.0
Station 8	s-8-1	1333.0	404.0	3768.0	4160.7	27.7
	s-8-2	1170.0	275.0	3123.0	3745.0	24.6
	s-8-3	1209.0	475.0	3230.0	3326.2	27.0

Mean and Std. Deviation of mineral content in 8 stations of Bandon Bay

Station		K	Ca	Mg	Na	Mn
**1**	Mean	2050.7	1279.7	3928.0	9134.9	691.7
	Std. Deviation	57.8	184.6	78.2	47.5	28.9
**2**	Mean	2671.7	4109.0	4547.0	6329.3	816.0
	Std. Deviation	40.0	938.9	347.6	272.3	23.4
**3**	Mean	3274.0	21860.0	6449.7	7364.9	976.7
	Std. Deviation	259.2	1130.1	431.4	191.3	225.5
**4**	Mean	2386.0	1514.3	3685.3	5407.7	629.7
	Std. Deviation	300.6	232.5	292.2	782.9	78.7
**5**	Mean	1436.7	1079.3	1978.3	4646.9	367.7
	Std. Deviation	60.2	148.8	47.3	144.8	56.2
**6**	Mean	2044.3	929.0	4016.0	8125.0	499.7
	Std. Deviation	16.3	78.3	904.0	2137.7	99.4
**7**	Mean	2857.0	513.0	6181.0	7143.1	723.0
	Std. Deviation	162.2	64.1	138.6	141.9	196.8
**8**	Mean	1237.3	384.7	3373.7	3744.0	26.4
	Std. Deviation	85.1	101.4	345.7	417.3	1.6

Data Analysis of mineral content in 8 stations

Anova

		Sum of Squares	df	Mean Square	F	Sig.
**K**	Between Groups	10146411.625	7	1449487.375	58.007	.000
	Within Groups	399807.333	16	24987.958		
	Total	10546218.958	23			
**Ca**	Between Groups	1127243030.958	7	161034718.708	562.679	.000
	Within Groups	4579082.667	16	286192.667		
	Total	1131822113.625	23			
**Mg**	Between Groups	45174183.958	7	6453454.851	38.056	.000
	Within Groups	2713244.667	16	169577.792		
	Total	47887428.625	23			
**Mn**	Between Groups	1816112.360	7	259444.623	18.841	.000
	Within Groups	220326.620	16	13770.414		
	Total	2036438.980	23			
**Na**	Between Groups	68986802.083	7	9855257.440	14.306	.000
	Within Groups	11021977.107	16	688873.569		
	Total	80008779.190	23			

Multiple Comparisons with Turkey HSD

Dependent Variable	(I) Station	(J) Station	Mean Difference (I-J)	Std. Error	Sig.	95% Confidence Interval	
						Lower Bound	Upper Bound
K	1	2	-621.0000*	129.0683	.004	-1067.854	-174.146
		3	-1223.3333*	129.0683	.000	-1670.187	-776.479
		4	-335.3333	129.0683	.226	-782.187	111.521
		5	614.0000*	129.0683	.004	167.146	1060.854
		6	6.3333	129.0683	1.000	-440.521	453.187
		7	-806.3333*	129.0683	.000	-1253.187	-359.479

		8	813.3333*	129.0683	.000	366.479	1260.187
	2	1	621.0000*	129.0683	.004	174.146	1067.854
		3	-602.3333*	129.0683	.005	-1049.187	-155.479
		4	285.6667	129.0683	.393	-161.187	732.521
		5	1235.0000*	129.0683	.000	788.146	1681.854
		6	627.3333*	129.0683	.003	180.479	1074.187
		7	-185.3333	129.0683	.828	-632.187	261.521
		8	1434.3333*	129.0683	.000	987.479	1881.187
	3	1	1223.3333*	129.0683	.000	776.479	1670.187
		2	602.3333*	129.0683	.005	155.479	1049.187
		4	888.0000*	129.0683	.000	441.146	1334.854
		5	1837.3333*	129.0683	.000	1390.479	2284.187
		6	1229.6667*	129.0683	.000	782.813	1676.521
		7	417.0000	129.0683	.077	-29.854	863.854
		8	2036.6667*	129.0683	.000	1589.813	2483.521
	4	1	335.3333	129.0683	.226	-111.521	782.187
		2	-285.6667	129.0683	.393	-732.521	161.187
		3	-888.0000*	129.0683	.000	-1334.854	-441.146
		5	949.3333*	129.0683	.000	502.479	1396.187
		6	341.6667	129.0683	.209	-105.187	788.521
		7	-471.0000*	129.0683	.035	-917.854	-24.146
		8	1148.6667*	129.0683	.000	701.813	1595.521
	5	1	-614.0000*	129.0683	.004	-1060.854	-167.146
		2	-1235.0000*	129.0683	.000	-1681.854	-788.146
		3	-1837.3333*	129.0683	.000	-2284.187	-1390.479
		4	-949.3333*	129.0683	.000	-1396.187	-502.479

		6	-607.6667*	129.0683	.005	-1054.521	-160.813
		7	-1420.3333*	129.0683	.000	-1867.187	-973.479
		8	199.3333	129.0683	.774	-247.521	646.187
	6	1	-6.3333	129.0683	1.000	-453.187	440.521
		2	-627.3333*	129.0683	.003	-1074.187	-180.479
		3	-1229.6667*	129.0683	.000	-1676.521	-782.813
		4	-341.6667	129.0683	.209	-788.521	105.187
		5	607.6667*	129.0683	.005	160.813	1054.521
		7	-812.6667*	129.0683	.000	-1259.521	-365.813
		8	807.0000*	129.0683	.000	360.146	1253.854
	7	1	806.3333*	129.0683	.000	359.479	1253.187
		2	185.3333	129.0683	.828	-261.521	632.187
		3	-417.0000	129.0683	.077	-863.854	29.854
		4	471.0000*	129.0683	.035	24.146	917.854
		5	1420.3333*	129.0683	.000	973.479	1867.187
		6	812.6667*	129.0683	.000	365.813	1259.521
		8	1619.6667*	129.0683	.000	1172.813	2066.521
	8	1	-813.3333*	129.0683	.000	-1260.187	-366.479
		2	-1434.3333*	129.0683	.000	-1881.187	-987.479
		3	-2036.6667*	129.0683	.000	-2483.521	-1589.813
		4	-1148.6667*	129.0683	.000	-1595.521	-701.813
		5	-199.3333	129.0683	.774	-646.187	247.521
		6	-807.0000*	129.0683	.000	-1253.854	-360.146
		7	-1619.6667*	129.0683	.000	-2066.521	-1172.813
Ca	1	2	-2829.3333*	436.8010	.000	-4341.604	-1317.062
		3	-20580.3333*	436.8010	.000	-22092.604	-19068.062

		4	-234.6667	436.8010	.999	-1746.938	1277.604
		5	200.3333	436.8010	1.000	-1311.938	1712.604
		6	350.6667	436.8010	.990	-1161.604	1862.938
		7	766.6667	436.8010	.655	-745.604	2278.938
		8	895.0000	436.8010	.483	-617.271	2407.271
	2	1	2829.3333*	436.8010	.000	1317.062	4341.604
		3	-17751.0000*	436.8010	.000	-19263.271	-16238.729
		4	2594.6667*	436.8010	.000	1082.396	4106.938
		5	3029.6667*	436.8010	.000	1517.396	4541.938
		6	3180.0000*	436.8010	.000	1667.729	4692.271
		7	3596.0000*	436.8010	.000	2083.729	5108.271
		8	3724.3333*	436.8010	.000	2212.062	5236.604
	3	1	20580.3333*	436.8010	.000	19068.062	22092.604
		2	17751.0000*	436.8010	.000	16238.729	19263.271
		4	20345.6667*	436.8010	.000	18833.396	21857.938
		5	20780.6667*	436.8010	.000	19268.396	22292.938
		6	20931.0000*	436.8010	.000	19418.729	22443.271
		7	21347.0000*	436.8010	.000	19834.729	22859.271
		8	21475.3333*	436.8010	.000	19963.062	22987.604
	4	1	234.6667	436.8010	.999	-1277.604	1746.938
		2	-2594.6667*	436.8010	.000	-4106.938	-1082.396
		3	-20345.6667*	436.8010	.000	-21857.938	-18833.396
		5	435.0000	436.8010	.968	-1077.271	1947.271
		6	585.3333	436.8010	.870	-926.938	2097.604
		7	1001.3333	436.8010	.354	-510.938	2513.604
		8	1129.6667	436.8010	.230	-382.604	2641.938
	5	1	-200.3333	436.8010	1.000	-1712.604	1311.938

	2	-3029.6667*	436.8010	.000	-4541.938	-1517.396
	3	-20780.6667*	436.8010	.000	-22292.938	-19268.396
	4	-435.0000	436.8010	.968	-1947.271	1077.271
	6	150.3333	436.8010	1.000	-1361.938	1662.604
	7	566.3333	436.8010	.887	-945.938	2078.604
	8	694.6667	436.8010	.749	-817.604	2206.938
6	1	-350.6667	436.8010	.990	-1862.938	1161.604
	2	-3180.0000*	436.8010	.000	-4692.271	-1667.729
	3	-20931.0000*	436.8010	.000	-22443.271	-19418.729
	4	-585.3333	436.8010	.870	-2097.604	926.938
	5	-150.3333	436.8010	1.000	-1662.604	1361.938
	7	416.0000	436.8010	.975	-1096.271	1928.271
	8	544.3333	436.8010	.906	-967.938	2056.604
7	1	-766.6667	436.8010	.655	-2278.938	745.604
	2	-3596.0000*	436.8010	.000	-5108.271	-2083.729
	3	-21347.0000*	436.8010	.000	-22859.271	-19834.729
	4	-1001.3333	436.8010	.354	-2513.604	510.938
	5	-566.3333	436.8010	.887	-2078.604	945.938
	6	-416.0000	436.8010	.975	-1928.271	1096.271
	8	128.3333	436.8010	1.000	-1383.938	1640.604
8	1	-895.0000	436.8010	.483	-2407.271	617.271
	2	-3724.3333*	436.8010	.000	-5236.604	-2212.062
	3	-21475.3333*	436.8010	.000	-22987.604	-19963.062
	4	-1129.6667	436.8010	.230	-2641.938	382.604
	5	-694.6667	436.8010	.749	-2206.938	817.604
	6	-544.3333	436.8010	.906	-2056.604	967.938
	7	-128.3333	436.8010	1.000	-1640.604	1383.938

Mg	1	2	-619.0000	336.2319	.604	-1783.085	545.085
		3	-2521.6667*	336.2319	.000	-3685.752	-1357.581
		4	242.6667	336.2319	.995	-921.419	1406.752
		5	1949.6667*	336.2319	.001	785.581	3113.752
		6	-88.0000	336.2319	1.000	-1252.085	1076.085
		7	-2253.0000*	336.2319	.000	-3417.085	-1088.915
		8	554.3333	336.2319	.717	-609.752	1718.419
	2	1	619.0000	336.2319	.604	-545.085	1783.085
		3	-1902.6667*	336.2319	.001	-3066.752	-738.581
		4	861.6667	336.2319	.238	-302.419	2025.752
		5	2568.6667*	336.2319	.000	1404.581	3732.752
		6	531.0000	336.2319	.755	-633.085	1695.085
		7	-1634.0000*	336.2319	.003	-2798.085	-469.915
		8	1173.3333*	336.2319	.047	9.248	2337.419
	3	1	2521.6667*	336.2319	.000	1357.581	3685.752
		2	1902.6667*	336.2319	.001	738.581	3066.752
		4	2764.3333*	336.2319	.000	1600.248	3928.419
		5	4471.3333*	336.2319	.000	3307.248	5635.419
		6	2433.6667*	336.2319	.000	1269.581	3597.752
		7	268.6667	336.2319	.991	-895.419	1432.752
		8	3076.0000*	336.2319	.000	1911.915	4240.085
	4	1	-242.6667	336.2319	.995	-1406.752	921.419
		2	-861.6667	336.2319	.238	-2025.752	302.419
		3	-2764.3333*	336.2319	.000	-3928.419	-1600.248
		5	1707.0000*	336.2319	.002	542.915	2871.085
		6	-330.6667	336.2319	.971	-1494.752	833.419
		7	-2495.6667*	336.2319	.000	-3659.752	-1331.581

		8	311.6667	336.2319	.979	-852.419	1475.752
	5	1	-1949.6667*	336.2319	.001	-3113.752	-785.581
		2	-2568.6667*	336.2319	.000	-3732.752	-1404.581
		3	-4471.3333*	336.2319	.000	-5635.419	-3307.248
		4	-1707.0000*	336.2319	.002	-2871.085	-542.915
		6	-2037.6667*	336.2319	.000	-3201.752	-873.581
		7	-4202.6667*	336.2319	.000	-5366.752	-3038.581
		8	-1395.3333*	336.2319	.013	-2559.419	-231.248
	6	1	88.0000	336.2319	1.000	-1076.085	1252.085
		2	-531.0000	336.2319	.755	-1695.085	633.085
		3	-2433.6667*	336.2319	.000	-3597.752	-1269.581
		4	330.6667	336.2319	.971	-833.419	1494.752
		5	2037.6667*	336.2319	.000	873.581	3201.752
		7	-2165.0000*	336.2319	.000	-3329.085	-1000.915
		8	642.3333	336.2319	.563	-521.752	1806.419
	7	1	2253.0000*	336.2319	.000	1088.915	3417.085
		2	1634.0000*	336.2319	.003	469.915	2798.085
		3	-268.6667	336.2319	.991	-1432.752	895.419
		4	2495.6667*	336.2319	.000	1331.581	3659.752
		5	4202.6667*	336.2319	.000	3038.581	5366.752
		6	2165.0000*	336.2319	.000	1000.915	3329.085
		8	2807.3333*	336.2319	.000	1643.248	3971.419
	8	1	-554.3333	336.2319	.717	-1718.419	609.752
		2	-1173.3333*	336.2319	.047	-2337.419	-9.248
		3	-3076.0000*	336.2319	.000	-4240.085	-1911.915
		4	-311.6667	336.2319	.979	-1475.752	852.419
		5	1395.3333*	336.2319	.013	231.248	2559.419

		6	-642.3333	336.2319	.563	-1806.419	521.752
		7	-2807.3333*	336.2319	.000	-3971.419	-1643.248
Mn	1	2	-124.3333	95.8138	.887	-456.055	207.388
		3	-285.0000	95.8138	.121	-616.722	46.722
		4	62.0000	95.8138	.997	-269.722	393.722
		5	324.0000	95.8138	.058	-7.722	655.722
		6	192.0000	95.8138	.508	-139.722	523.722
		7	-31.3333	95.8138	1.000	-363.055	300.388
		8	665.2333*	95.8138	.000	333.512	996.955
	2	1	124.3333	95.8138	.887	-207.388	456.055
		3	-160.6667	95.8138	.701	-492.388	171.055
		4	186.3333	95.8138	.543	-145.388	518.055
		5	448.3333*	95.8138	.005	116.612	780.055
		6	316.3333	95.8138	.067	-15.388	648.055
		7	93.0000	95.8138	.972	-238.722	424.722
		8	789.5667*	95.8138	.000	457.845	1121.288
	3	1	285.0000	95.8138	.121	-46.722	616.722
		2	160.6667	95.8138	.701	-171.055	492.388
		4	347.0000*	95.8138	.037	15.278	678.722
		5	609.0000*	95.8138	.000	277.278	940.722
		6	477.0000*	95.8138	.003	145.278	808.722
		7	253.6667	95.8138	.209	-78.055	585.388
		8	950.2333*	95.8138	.000	618.512	1281.955
	4	1	-62.0000	95.8138	.997	-393.722	269.722
		2	-186.3333	95.8138	.543	-518.055	145.388
		3	-347.0000*	95.8138	.037	-678.722	-15.278
		5	262.0000	95.8138	.181	-69.722	593.722

		6	130.0000	95.8138	.863	-201.722	461.722
		7	-93.3333	95.8138	.972	-425.055	238.388
		8	603.2333*	95.8138	.000	271.512	934.955
	5	1	-324.0000	95.8138	.058	-655.722	7.722
		2	-448.3333*	95.8138	.005	-780.055	-116.612
		3	-609.0000*	95.8138	.000	-940.722	-277.278
		4	-262.0000	95.8138	.181	-593.722	69.722
		6	-132.0000	95.8138	.855	-463.722	199.722
		7	-355.3333*	95.8138	.031	-687.055	-23.612
		8	341.2333*	95.8138	.041	9.512	672.955
	6	1	-192.0000	95.8138	.508	-523.722	139.722
		2	-316.3333	95.8138	.067	-648.055	15.388
		3	-477.0000*	95.8138	.003	-808.722	-145.278
		4	-130.0000	95.8138	.863	-461.722	201.722
		5	132.0000	95.8138	.855	-199.722	463.722
		7	-223.3333	95.8138	.335	-555.055	108.388
		8	473.2333*	95.8138	.003	141.512	804.955
	7	1	31.3333	95.8138	1.000	-300.388	363.055
		2	-93.0000	95.8138	.972	-424.722	238.722
		3	-253.6667	95.8138	.209	-585.388	78.055
		4	93.3333	95.8138	.972	-238.388	425.055
		5	355.3333*	95.8138	.031	23.612	687.055
		6	223.3333	95.8138	.335	-108.388	555.055
		8	696.5667*	95.8138	.000	364.845	1028.288
	8	1	-665.2333*	95.8138	.000	-996.955	-333.512
		2	-789.5667*	95.8138	.000	-1121.288	-457.845
		3	-950.2333*	95.8138	.000	-1281.955	-618.512

		4	-603.2333*	95.8138	.000	-934.955	-271.512
		5	-341.2333*	95.8138	.041	-672.955	-9.512
		6	-473.2333*	95.8138	.003	-804.955	-141.512
		7	-696.5667*	95.8138	.000	-1028.288	-364.845
Na	1	2	2805.6000*	677.6792	.014	459.373	5151.827
		3	1770.0667	677.6792	.221	-576.161	4116.294
		4	3727.2000*	677.6792	.001	1380.973	6073.427
		5	4488.0667*	677.6792	.000	2141.839	6834.294
		6	1009.9667	677.6792	.802	-1336.261	3356.194
		7	1991.8333	677.6792	.129	-354.394	4338.061
		8	5390.9667*	677.6792	.000	3044.739	7737.194
	2	1	-2805.6000*	677.6792	.014	-5151.827	-459.373
		3	-1035.5333	677.6792	.783	-3381.761	1310.694
		4	921.6000	677.6792	.862	-1424.627	3267.827
		5	1682.4667	677.6792	.269	-663.761	4028.694
		6	-1795.6333	677.6792	.208	-4141.861	550.594
		7	-813.7667	677.6792	.920	-3159.994	1532.461
		8	2585.3667*	677.6792	.026	239.139	4931.594
	3	1	-1770.0667	677.6792	.221	-4116.294	576.161
		2	1035.5333	677.6792	.783	-1310.694	3381.761
		4	1957.1333	677.6792	.140	-389.094	4303.361
		5	2718.0000*	677.6792	.018	371.773	5064.227
		6	-760.1000	677.6792	.942	-3106.327	1586.127
		7	221.7667	677.6792	1.000	-2124.461	2567.994
		8	3620.9000*	677.6792	.001	1274.673	5967.127
	4	1	-3727.2000*	677.6792	.001	-6073.427	-1380.973

		2	-921.6000	677.6792	.862	-3267.827	1424.627
		3	-1957.1333	677.6792	.140	-4303.361	389.094
		5	760.8667	677.6792	.942	-1585.361	3107.094
		6	-2717.2333*	677.6792	.018	-5063.461	-371.006
		7	-1735.3667	677.6792	.239	-4081.594	610.861
		8	1663.7667	677.6792	.281	-682.461	4009.994
	5	1	-4488.0667*	677.6792	.000	-6834.294	-2141.839
		2	-1682.4667	677.6792	.269	-4028.694	663.761
		3	-2718.0000*	677.6792	.018	-5064.227	-371.773
		4	-760.8667	677.6792	.942	-3107.094	1585.361
		6	-3478.1000*	677.6792	.002	-5824.327	-1131.873
		7	-2496.2333*	677.6792	.033	-4842.461	-150.006
		8	902.9000	677.6792	.873	-1443.327	3249.127
	6	1	-1009.9667	677.6792	.802	-3356.194	1336.261
		2	1795.6333	677.6792	.208	-550.594	4141.861
		3	760.1000	677.6792	.942	-1586.127	3106.327
		4	2717.2333*	677.6792	.018	371.006	5063.461
		5	3478.1000*	677.6792	.002	1131.873	5824.327
		7	981.8667	677.6792	.822	-1364.361	3328.094
		8	4381.0000*	677.6792	.000	2034.773	6727.227
	7	1	-1991.8333	677.6792	.129	-4338.061	354.394
		2	813.7667	677.6792	.920	-1532.461	3159.994
		3	-221.7667	677.6792	1.000	-2567.994	2124.461
		4	1735.3667	677.6792	.239	-610.861	4081.594
		5	2496.2333*	677.6792	.033	150.006	4842.461
		6	-981.8667	677.6792	.822	-3328.094	1364.361

		8	3399.1333*	677.6792	.002	1052.906	5745.361
	8	1	-5390.9667*	677.6792	.000	-7737.194	-3044.739
		2	-2585.3667*	677.6792	.026	-4931.594	-239.139
		3	-3620.9000*	677.6792	.001	-5967.127	-1274.673
		4	-1663.7667	677.6792	.281	-4009.994	682.461
		5	-902.9000	677.6792	.873	-3249.127	1443.327
		6	-4381.0000*	677.6792	.000	-6727.227	-2034.773
		7	-3399.1333*	677.6792	.002	-5745.361	-1052.906

*. The mean difference is significant at the 0.05 level.
